# Cytochrome P450 Genes Expressed in Phasmatodea Midguts

**DOI:** 10.3390/insects13100873

**Published:** 2022-09-26

**Authors:** Matan Shelomi

**Affiliations:** Department of Entomology, National Taiwan University, Taipei City 10617, Taiwan; mshelomi@ntu.edu.tw; Tel.: +886-02-33665588

**Keywords:** cytochrome P450, Phasmatodea, stick insects, xenobiotic metabolism

## Abstract

**Simple Summary:**

Cytochrome P450s are a group of detoxification enzymes found in all animals. They are highly diverse, with multiple copies of different families of these genes in individual organisms, especially those that need to digest chemically-defended foods, such as toxic plants. The stick insects are a group of leaf-feeding herbivores whose diets can include toxic species, such as *Eucalyptus*, so this study sought to identify what groups of cytochrome P450s are expressed in the digestive tracts of six species of stick insects and how they evolved. The results show a complement of detoxification genes comparable to or slightly more limited than those of other insects, except for multiple enzymes per species in the subfamily *CYP15A1*, while most insects will only have one enzyme in this group. The functions of these expanded enzyme groups should be investigated further.

**Abstract:**

Cytochrome P450s (*CYP*s) are xenobiotic detoxification genes found in most eukaryotes, and linked in insects to the tolerance of plant secondary chemicals and insecticide resistance. The number and diversity of *CYP* clans, families, and subfamilies that an organism produces could correlate with its dietary breadth or specialization. This study examined the *CYP* diversity expressed in the midguts of six species of folivorous stick insects (Phasmatodea), to identify their *CYP* complement and see if any *CYP*s correlate with diet toxicity or specialization, and see what factors influenced their evolution in this insect order. *CYP* genes were mined from six published Phasmatodea transcriptomes and analyzed phylogenetically. The Phasmatodea *CYP* complement resembles that of other insects, though with relatively low numbers, and with significant expansions in the *CYP* clades *6J1*, *6A13/14*, *4C1*, and *15A1*. The *CYP*6 group is known to be the dominant *CYP* family in insects, but most insects have no more than one *CYP15* gene, so the function of the multiple *CYP15A1* genes in Phasmatodea is unknown, with neofunctionalization following gene duplication hypothesized. No correlation was found between *CYP*s and diet specialization or toxicity, with some *CYP* clades expanding within the Phasmatodea and others likely inherited from a common ancestor.

## 1. Introduction

Many plants are chemically defended against herbivores, producing directly toxic or anti-nutritive chemicals. Different herbivores use different strategies to overcome these. Some have symbiotic microbes that can metabolize and deactivate ingested toxins, while others produce their own endogenous detoxification compounds, and some have both [1]. Xenobiotic detoxification proteins in insects include glutathione-S-transferases (*GST*s) [2], carboxy/cholinesterases (*CE*s) and cytochrome P450 monooxygenases (*CYP*s). The number of such genes in different insect species varies, and they are often expressed in different tissues performing different functions [3] and/or in different life stages of the host [4]. These detoxification genes are linked to insecticide resistance [5], and so are of significant interest in applied entomology and other industries [6], yet are also informative regarding the evolution of dietary specification. Organisms that specialize in a particularly toxic plant would need to have evolved a powerful detoxification method in order to maintain that niche. Conversely, organisms with broad host and/or geographic ranges may need expanded xenobiotic metabolism genes to cover the wider variety of compounds they may be exposed to [7].

The Cytochrome P450s are found in all animals and plants and most microbes. Their high diversity in Metazoa is thought to be a direct result of herbivore–plant warfare [8], and the *CYP*s are notorious for high levels of gene duplication, transposition, neofunctionalization, and pseudogene development [9,10]. The present nomenclature for *CYP*s is *CYP#X#*, with the first numeral for the *CYP* family, the letter for the subfamily, and the second numeral for the gene [11]. By convention, a *CYP* family is defined as sharing > 40% sequence similarity along its members’ approximately 500 amino acids [12]. *CYP*s are found in all insect tissues. Above the family level, insect *CYP*s are sorted into four clans: the mitochondrial clan, clan 2, clan 3, and clan 4, with clan 3 considered most important for xenobiotic regulation while the others are involved in conserved physiological roles such as hormone synthesis [3,11,13]. The mitochondrial clan is nuclear-encoded and named because of their mitochondria-targeting sequences [14] The number of *CYP* genes in insects varies: some parasitoids have less than 30 [5], while mosquitoes have over 200 [13,15]. Those expressed in the midgut, Malpighian tubules, and fat body are integral for plant chemical detoxification and insecticide resistance [16,17], as confirmed by heterologous expression and transcriptomics studies [4,12].

The Phasmatodea, or leaf and stick insects, are an order of obligatorily herbivorous insects in the Polyneoptera clade. They are not known to have microbial symbionts and process their food with endogenous digestive and detoxification proteins [18,19]. Several are of economic significance [20]. *Graeffea crouanii* (Le Guillou 1841) is a pest of coconut plantations in the south Pacific [21]. Outbreaks of *Diapheromera femorata* (Say 1924) (Diapheromeridae) in the USA can cause severe forest defoliation [22]. In Australia, plague-like outbreaks of *Didymuria violescens* Leach 1815 (Phasmatidae), *Anchiale austrotessulata* Brock & Hasenpusch 2007 (Phasmatidae), and *Podacanthus wilkinsoni* Macleay 1882 (Phasmatidae) can cause severe defoliation to *Eucalyptus* L’Her. trees [23]. The latter is particularly remarkable given how toxic *Eucalyptus* is [24]. Understanding how these species evolved the ability to metabolize, eliminate, or otherwise endure *Eucalyptus* secondary compounds has basic and applied science significance. 

This study compared the *CYP*s expressed in the digestive tracts of six different Phasmatodea species, representing four families and five subfamilies and fed three different species of the host plant, to understand how *CYP*s evolved within Phasmatodea and attempt to identify *CYP* families that may play important roles in plant chemical detoxification.

## 2. Materials and Methods

This study uses published Phasmatodea transcriptome data (GenBank Accession numbers PRJNA238833 and PRJNA221630) from the physiologically distinct anterior and posterior midguts [19] of *Aretaon asperrimus* (Redtenbacher 1906) (Heteropterygidae: Obriminae), *Extatosoma tiaratum* (Macleay 1826) (Phasmatidae: Extatosomatinae), *Medauroidea extradentata* Brunner von Wattenwyl 1907 (Phasmatidae: Clitumninae), *Peruphasma schultei* Conle & Hennemann 2005 (Pseudophasmatidae), *Ramulus artemis* (Westwood 1859) (Phasmatidae: Clitumninae), and the anterior midgut only of *Sipyloidea sipylus* (Westwood 1859) (Lonchodidae) [25]. The *Extatosoma* were reared on *Eucalyptus* sp., the *Peruphasma* were reared on privet (*Ligustrum* sp. L.), and the others fed on rose leaves (*Rosa* sp. L.). 

In June 2021, representative sequences for insect cytochrome P450s were downloaded from NCBI, limiting the search to those in the UniProtKB database [26,27]. The resulting 111 sequences were used as a query to mine the above transcriptomes using tblastn [28] with an expected value threshold of e−10. These were manually annotated by removing truncated sequences, using the ExPASy online translation tool [29] to obtain the complete amino acid sequences, removing duplicates using the sRNA toolbox webserver [30], and confirming that the sequences were cytochrome P450s by identifying them using blastp against the NCBI database. This search also enabled the identification of the sequences to the *CYP* family: according to standard *CYP* nomenclature, sequences with more than 40% identity are in the same family [31]. The resulting sequences were combined with the representatives from NCBI, aligned using the Clustal W program [32] built into the software MEGA version X [33]. Any sequences missing the heme-binding domain FXXGXXXCXG/A [34], which is a signature motif for *CYP*s [6], were deleted. The sequences were also checked for the presence and absence of other four signature motifs from insect *CYP*s: helix C (WxxxR), helix I (GxE/DTT/S), helix K (ExLR), and PERF (PxxFxPE/DRE/F) [35]. Fasta files for the amino acid sequences of these putative *CYP*s from the six transcriptomes were uploaded to Zenodo (DOI: 10.5281/zenodo.7049585).

All validated Phasmatodea species’ *CYP*s and select insect *CYP*s from the NCBI/UniProt databases were aligned using Clustal Omega [36], and phylogenetic analysis was done using the neighbor-joining method [37] with 10,000 bootstraps in MEGA version X [33], to confirm and if necessary correct the identities assigned to each sequence via blastp. This tree would also show if Phasmatodea genes that have multiple copies in one species also have multiple copies in another, which could help time any gene duplication or expansion events across Phasmatodea evolutionary history. Phylogenetic tree inference using maximum likelihood with rapid bootstrapping was made using the IQ-TREE web server [38] with 1000 ultrafast bootstrapping replicates [39] and the best-fitting model LG+I+G4 as identified using ModelFinder [40]. The tree was visualized using FigTree v1.4.4.

## 3. Results

Table 1 lists the number of isogroups (comp#_c#) and isotigs (comp#_c#_seq#, or individual transcript sequences) of each identifiable *CYP* family in each of the Phasmatodea, limiting the results to non-truncated sequences. All valid sequences containing the heme-binding domain had a blastp sequence similarity > 40% to a known *CYP* subfamily; however, some sequences were equally similar to known *6A13* and *6A14* genes, or to the *4C1* and *4C3* genes. Most *CYP* families had a single gene per species, while a few were highly expanded: *6J1*, *6A13/14*, *4C1*, and *15A1*. *Medauroidea* had the largest number of *CYP* transcripts, followed by the other two Phasmatidae species, *Ramulus* and *Extatosoma*, largely due to isogroup and isotig expansions in the *6J1* family. *Peruphasma* also had many *6J1* isotigs, but few of the others. *Aretaon* had comparatively many *15A1* transcripts but the fewest 6J1. *Sipyloidea* had the fewest isotigs and tied with *Peruphasma* for the fewest isogroups. The *Sipyloidea* transcriptome had zero representatives from the mitochondrial clan, though *Peruphasma* and *Extatosoma* each only had two.

**Figure 1:**
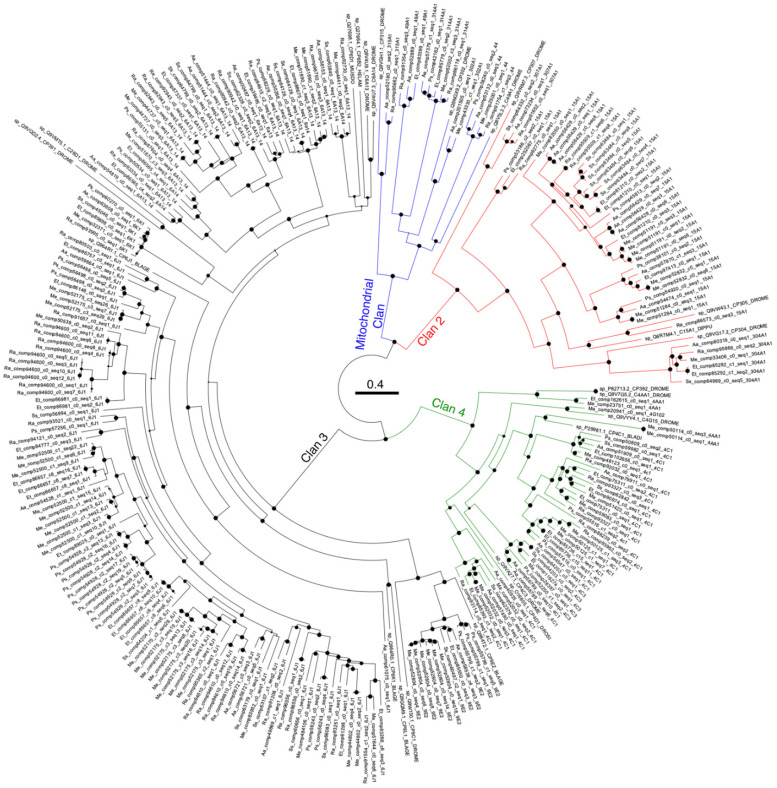
Maximum-likelihood phylogenetic tree of the Phasmatodea *CYP450s*. The evolutionary history was inferred using the Maximum likelihood method with the model LG+I+G4, identified as the best via Bayesian information criterion scores. The consensus tree with the log-likelihood −129,334.593 is shown, with the sum of branch lengths 72.0129. The percentage of replicate trees in which the associated taxa clustered together in the bootstrap test (1000 replicates) are depicted as ten possible sizes of circles at the nodes, with the largest nodes indicating 100% [41]. The tree is drawn to scale, with branch lengths in the same units as those of the evolutionary distances used to infer the phylogenetic tree. The tree was edited using FigTree v 1.4.4 and Adobe Photoshop. Phasmatodea *CYP* isotigs are labeled with a two-letter code for the species (*Aa* = *Aretaon asperrimus*. *Et* = *Extatosoma tiaratum*. *Me* = *Medauroidea extradentata*. *Ps* = *Peruphasma schultei*. *Ra* = *Ramulus artemis*. *Ss* = *Sipyloidea sipylus*) followed by the Trinity [42] output comp#_c#_seq_#, then a “_” and the putative *CYP* family, subfamily, and gene in #X# format. Sequences from GenBank are labeled sp_[Genbank Accession number]|CP#x#_[UniProt organism code]. The *CYP* clans are labeled: Mito[chondrial] clan in blue, Clan 2 in red, Clan 3 in black, and Clan 4 in green. Credit: M. Shelomi.

Figure 1 shows the maximum likelihood tree of the representative *CYP* isogroups from Phasmatodea and other insects. The *CYP* clans form well-supported monophyletic groups, and the tree clusters according to the *CYP* family rather than insect taxonomy. Most isogroups had one representative from each Phasmatodea, with *Peruphasma* and *Sipyloidea* the most frequent missing species. Any isogroups expanded in one species were typically also expanded in another, with isotigs clustering according to species.

Modifications of motifs clustered by *CYP* subfamily, not by species. *CYP307* is missing helix C completely, and one monophyletic clade of Phasmatodea *CYPA13*/*14* has the motif as WxxxK instead of WxxxR. Helix I (GxE/DTT/S) is completely absent in *CYP315* and *CYP304*, including the non-Phasmatodea sequence. It is mutated as GxDxT in *CYP49*, GxEST or GxE/DTx in the Phasmatodea *CYP15A*’s, GxxTT in *CYP44*, GxxxT in *CYP314*, GxE/Dxx in *CYP4AA* and *CYP392*, and Gxxxx in *CYP307*. Helix K mutations were widespread, especially in the *CYP15A* and *CYP6K* subfamilies. The PERF motif was mostly conserved with scattered exceptions.

## 4. Discussion

It is not always possible to tell whether individual transcripts/isotigs represent truly different genes, different alleles from different individuals, splice variations, or errors in assembling the original transcriptomes. However, the original study compared the *Peruphasma schultei* transcriptome data to genome data from the same individuals and concluded that the isotigs represent true isoforms within each isogroup, and thus are closer to the actual gene number [25]. The numbers identified from this analysis are still likely an underestimate of the total number of *CYP* genes for each species, as only midguts were sampled, and all from insects that had not been given a toxin, pathogen, or stress challenge that could induce expression of different *CYP*s. As the original study was unable to produce a transcriptome from the posterior midgut of *Sipyloidea sipylus*, it is likely that some *CYP*s differentially expressed in that subregion of the midgut are missing from that species’ dataset: probable examples based on Table 1 are the *44*, *314A1*, and some *4C1* and *15A1 CYP*s. Moreover, as 5′ and 3′ truncated transcripts were deleted, some misassembled genes would not be in this dataset, so the isotig numbers at least are likely underestimates. These caveats do not affect the general conclusions of this study or the ability of the data to test the qualitative hypotheses of this study, and only mean the quantitative numbers in Table 1 are meant to be preliminary rather than definitive. 

*Peruphasma* and *Sipyloidea* each had 19 different *CYP* isogroups, while the others had 27–33. Some isogroups had multiple paralogous isotigs, and such isogroups in one species would also have paralogues in another, suggesting the gene duplications occurred in a common ancestor of these six species. The low isogroup number in *Sipyloidea* likely reflects the lack of posterior midgut tissue that includes the Phasmatodea-specific excretory organs, the “appendices of the midgut,” which are known to have differentially expressed *CYP*s and play a role in xenobiotic metabolism [43]. Why the mitochondrial clan *CYP*s are expressed in the posterior midgut but not the anterior midgut is worth examining further. If one assumes that a *Sipyloidea* transcriptome that included the posterior midgut would have higher transcript numbers, then the relatively low isogroup number in *Peruphasma* is likely due to it being in a separate lineage from the others: a recent Phasmatodea phylogenomic work separated the New World Occidophasmata, which includes the Peruvian *Peruphasma*, and the Old World Oriophasmata that includes the other five species [44]. The expansions within the different *CYP* families do not all match this phylogeny. Some of the *6A13/14* and *15A1 CYP* branches show higher diversity among the Oriophasmata, or have the *P. shcultei* gene as a sister to the others. The *6J1*s show many duplications that occurred after the individual species evolved, not before. As most recent phylogenomic analyses of the Phasmatodea disagree on the relative placements of the non-Phasmatidae families [44,45,46], one cannot currently be certain of where the expansions of these *CYP* families occurred. For example, recent studies placed *P. schultei* as more closely related to *Medauroidea*, *Ramulus*, and *Extatosoma*, with *Aretaon* and *Sipyloidea* as more basal groups [45,47]. From Figure 1, this type of branching is seen in the *CYP* group *304A1*, but not in the clan 4 *CYP*s, for example, though the low levels of *Sipyloidea CYP* transcripts affect the data. Ultimately one cannot currently approach the different Phasmatodea phylogenetic hypotheses using *CYP* expansion data.

As in the majority of insects [3,12,15,48], the Phasmatodea *CYP*s are mostly in the *CYP6* family. This clan 3 family unique to insects is accepted as their primary xenobiotic metabolism *CYP*. *CYP6J* and *CYP6A* seem to be the conserved, dominant *CYP* subfamilies in the Phasmatodea, inherited from a common ancestor but with frequent gene duplication events that could reflect neofunctionalizations. Differentiating between *CYP6J1*, *6A13*, and *6A14* is difficult, and these three genes and other *CYP*6′s are closely related as evidenced in Figure 1 and the literature [49,50]. All Phasmatodea also expressed a single *CYP6K* inherited from a common ancestor. *CYP6J1* and *CYP6K1* were originally described as microsomal *CYP*s in the cockroach *Blatella germanica* (L.), where they are expressed in all life stages but at higher levels in the adult abdomen, but their function was not speculated [49]. *CYP6D1* affects pyrethroid resistance in the housefly *Musca domestica* L. 1758, while *CYP6B*’s in *Papilio* L. 1758 butterflies detoxify plant furanocoumarins [4]. *CYP6G1* expression in the *Drosophila* Malpighian tubules directly correlates with DDT resistance [17]. Knockdown of four different *CYP6* genes in *Locusta migratoria* L. 1758 increased nymph mortality following insecticide exposure [51]. A CRISPR-Cas9 knockout of *CYP6AE* in the extremely polyphagous *Helicoverpa armigera* (Hübner 1808) reduced its resistance to both host plant chemicals and insecticides [52], with similar results obtained from RNAi knockdown of *CYP6AB60* in the cutworm *Spodoptera litura* (Fabricius 1775) [53]. 

*CYP4*′s are in *CYP* clan 4, and produce isozymes linked to xenobiotic metabolism as well as odorant metabolism [3]. *CYP4C1* is one of the earliest known *CYP*s from insects [12], and is induced by endogenous hypertrehalosemic hormone [4]. Most research on this gene focuses on its relationship to hormones, not xenobiotics [54]. In addition to *CYP4C*, some but not all Phasmatodea also expressed *CYP4AA* and *CYP4G* genes. *CYP15A* is in *CYP* clan 2, which is primarily involved in basic physiological functions. *CYP15A1* in particular is involved in juvenile hormone metabolism, specifically the epoxidation of methyl farnesoate to juvenile hormone III [55,56]. 

The other families, with the exception of *4AA1* in *Medauroidea*, had no more than one representative isogroup per species and most only had one isotig each. Even assuming that these numbers are underestimates, it is still unlikely that less conservative criteria for including and excluding sequences in the final analysis would have produced more hits for these families: these Phasmatodea likely inherited one copy of each gene from their common ancestor. These families include several from the mitochondrial clan of *CYP*s, including *44*, *49A*, *302A*, *314A*, and *315A*, which are highly conserved and essential for physiological functions such as molting and development [3,11,57]. *CYP304* and *CYP307* are in clan 2 with *CYP15*, and also hold non-defensive functions, such as ecdysteroid synthesis [3]. *CYP9* is in clan 3 along with *CYP6* and is also involved in xenobiotic metabolism [3].

The Phasmatodea *CYP* complement can be compared to those of other insects. A study of the *Plutella xylostella* (L.) genome similarly found many copies of *CYP6*, albeit from different subfamilies, but also found many genes of *CYP340*, which is a clan 4 *CYP* associated with xenobiotic detoxification, multiple *CYP*9 genes, and only one *CYP15* gene [3]. The *Drosophila* genome contains 86 *CYP* genes in 25 families, more than half of which were *CYP4* and *CYP6* [58]. An RNA-Seq study of the polyphagous stink bug *Halyomorpha halys* Stål 1855 found a minimum 163 *CYP* genes, of which 105 were in clan 3, 46 in clan 4, and six each in clan 2 and the mitochondrial clan. The *H. halys CYP6*s were primarily in the B subfamily, and only one *CYP15* gene was found [13]. Genome analysis of the mosquito *Aedes aegypti* found 44 *CYP6* and 37 *CYP9* genes, and only one *CYP15* [15]. That only one *CYP9* gene was ever found in the Phasmatodea midgut transcriptomes despite this family being expanded in other insects could suggest the gene family is more highly expressed in other tissues not sampled in the original transcriptome study, such as the ‘fat body’. It is not clear why Phasmatodea have so many more *CYP15* genes than other insects: there seems to be no precedent for an insect with multiple *CYP15A1* genes, nor is this juvenile hormone epoxidase gene commonly associated with the midgut [56]. Recall that *CYP* pseudogenes are relatively common, so the possibility exists that some of these transcripts are for nonfunctional proteins [9]. The functions of the Phasmatodea *CYP15A1*s are for now an open mystery.

*Extatosoma tiaratum* was particularly interesting for this study due to its ability to eat *Eucalyptus*. *CYP*s play roles in detoxifying *Eucalyptus* in marsupial mammals, such as *CYP2C* and *CYP4A* in the koala, *Phascolarctos cinereus* (Goldfuss 1817) [59,60]. No *CYP* clade was particularly expanded in *Extatosoma* but not in other phasmids, so one cannot presently speculate which of the *Extatosoma CYP*s is most important for *Eucalyptus* detoxification. That ‘question’ could be answered with an RNA-Seq experiment comparing transcriptomes of *Extatosoma tiaratum* reared on *Eucalyptus* and those reared on another suitable diet, such as *Rosa.* One should also note that *E. tiaratum* is not a *Eucalyptus* specialist, but rather a polyphagous species that happens to also eat *Eucalyptus* in the wild. Comparing *Extatosoma* with other *Eucalyptus*-feeding insects, particularly specialists and including but not limited to other Phasmatodea, would be informative. Presently we see no evidence from the Phasmatodea data that either toxic diets or polyphagy correlate with *CYP* diversity.

The other Phasmatodea with a highly chemically defended diet, *Peruphasma*, similarly did not show any expansion in any *CYP* families relative to other organisms. Their natural food plant is *Schinus molle*, the Peruvian pepper or *pirul*, whose leaf essential oils are known insecticides [61], but it remains unknown how exactly *P. schultei* survives such a toxic diet. Their food plant in captivity, *Ligustrum*, produces an iridoid glycoside, oleuropein, which decreases lysine levels and makes the plant proteins non-nutritious. Metabolizing oleuropein with a *CYP* is not the only means of nullifying it, nor is it a common strategy. Lepidoptera and Hymenoptera that specialize in *Ligustrum* secrete large amounts of glycine or, less often, GABA or β-alanine, into their gut that counteract the lysine-eliminating effect [62]. *Peruphasma* may use a similar strategy, though this hypothesis has yet to be tested. 

## 5. Conclusions

In conclusion, the Phasmatodea have conserved, orthologous *CYP*s likely inherited from a common ancestor, with the likely primary xenobiotic-detoxification proteins in the *CYP6J* and *CYP6A* families. Within those families, typically one or two isogroups had an unusually large number of isotigs, suggesting gene duplication events, splice variants, or other modifications that in turn suggest possible neofunctionalization as observed with Phasmatodea digestive enzymes [63]. These would be the primary genes to study in any future knockdown experiments. The curious expansion in *CYP15A1* needs to be investigated further, with hypothesized explanations including neofunctionalization of xenobiotic metabolism ability or pseudogenes: the sequences with missing or mutated *CYP* conserved motifs may or may not be examples thereof. The Phasmatodea gut *CYP* arsenal seems relatively limited. While many species are oligo- or polyphagous, individual insects often prefer to feed on the same species they first fed on [64], so it is possible that the Phasmatodea genomes may contain more *CYP* genes than need to be expressed by any individual over its lifetime. However, as other authors have noted, it is difficult to draw conclusions about diets from comparative *CYP* expressions in different species, even within the same genus or insect family [15]. *CYP*s in an organism likely reflect its evolutionary origin more than its diet, although broader studies are needed to confirm this.

## Figures and Tables

**Table 1:** Cytochrome P450s of the Phasmatodea.

		Isogroups (Isotigs)					
*CYP450* Clan	Family	*Aa*	*Et*	*Me*	*Ps*	*Ra*	Ss *
Mito. Clan	*44*	1 (1)	0	1 (1)	1 (1)	1 (1)	0
	*49A1*	1 (1)	1 (1)	0	0	1 (1)	0
	*302A1*	1 (1)	0	1 (1)	0	0	0
	*314A1*	1 (1)	1 (1)	1 (1)	1 (1)	1 (1)	0
	*315A1*	1 (1)	0	0	0	1 (1)	0
Clan 2	*304A1*	1 (1)	1 (2)	1 (1)	0	1 (1)	1 (1)
	*307A1*	1 (1)	0	0	0	1 (1)	1 (1)
	*15A1*	3 (8)	3 (5)	4 (9)	3 (3)	3 (4)	1 (6)
Clan 3	*6J1*	5 (6)	8 (15)	7 (24)	4 (16)	10 (25)	6 (6)
	*6A13/14*	5 (6)	4 (5)	4 (6)	3 (3)	7 (10)	4 (6)
	*6K1*	1 (1)	1 (1)	1 (1)	1 (1)	1 (1)	1 (1)
	*9E2*	1 (1)	1 (1)	1 (6)	1 (3)	0	1 (1)
Clan 4	*4AA1*	0	1 (1)	2 (2)	0	0	0
	*4C1*	6 (7)	6 (7)	6 (9)	4 (4)	5 (5)	4 (5)
	*4G102*	0	0	1 (1)	0	0	0

Isogroup names are based on the original transcriptome data. Values in parentheses are the number of isotigs per isogroup if >1. * Transcriptome based on the anterior midgut only, while other species’ transcriptomes are the complete anterior and posterior midgut. *Aa* = *Aretaon asperrimus*. *Et* = *Extatosoma tiaratum*. *Me* = *Medauroidea extradentata*. *Ps* = *Peruphasma schultei*. *Ra* = *Ramulus artemis*. *Ss* = *Sipyloidea sipylus*.

## Data Availability

Publicly archived data (the amino acid sequences used for the phylogenetic trees) were uploaded to Zenodo and are available at https://doi.org/10.5281/zenodo.7049585.
